# Cost and outcomes of routine HIV care and treatment: public and private service delivery models covering low-income earners in South Africa

**DOI:** 10.1186/s12913-023-09147-7

**Published:** 2023-03-11

**Authors:** L. C. Long, S. Girdwood, K. Govender, G. Meyer-Rath, J. Miot

**Affiliations:** 1 Health Economics and Epidemiology Research Office (HE²RO), Faculty of Health Sciences, University of Witwatersrand, Johannesburg, South Africa; 2 Department of Global Health, Boston University School of Public Health, Boston, United States

**Keywords:** South Africa, HIV, Private sector, Antiretroviral therapy, Cost and cost analysis

## Abstract

**Background:**

While South Africa’s national HIV program is the largest in the world, it has yet to reach the UNAIDS 95–95-95 targets. To reach these targets, the expansion of the HIV treatment program may be accelerated through the use private sector delivery models. This study identified three innovative non-governmental primary health care models (private sector) providing HIV treatment, as well as two government primary health clinics (public sector) that served similar populations. We estimated the resources used, and costs and outcomes of HIV treatment across these models to provide inputs to inform decisions around how these services might best be provided through National Health Insurance (NHI).

**Methods:**

A review of potential private sector models for HIV treatment in a primary health care setting was conducted. Models actively offering HIV treatment (i.e. in 2019) were considered for inclusion in the evaluation, subject to data availability and location. These models were augmented by government primary health clinics offering HIV services in similar locations. We conducted a cost-outcomes analysis by collecting patient-level resource usage and treatment outcomes through retrospective medical record reviews and a bottom-up micro-costing from the provider perspective (public or private payer). Patient outcomes were based on whether the patient was still in care at the end of the follow up period and viral load (VL) status, to create the following outcome categories: in care and responding (VL suppressed), in care and not responding (VL unsuppressed), in care (VL unknown) and not in care (LTFU or deceased). Data collection was conducted in 2019 and reflects services provided during the 4 years prior to that (2016–2019).

**Results:**

Three hundred seventy-six patients were included across the five HIV treatment models. Across the three private sector models there were differences in the costs and outcomes of HIV treatment delivery, two of the models had results similar to the public sector primary health clinics. The nurse-led model appears to have a cost-outcome profile distinct from the others.

**Conclusion:**

The results show that across the private sector models studied the costs and outcomes of HIV treatment delivery vary, yet there were models that provided costs and outcomes similar to those found with public sector delivery. Offering HIV treatment under NHI through private delivery models could therefore be an option to increase access beyond the current public sector capacity.

## Introduction

Globally, UNAIDS has set the 95–95-95 targets for HIV testing, treatment and viral suppression by 2030 [1]. While South Africa’s national HIV program is the largest in the world and continues to grow, it has yet to reach these targets [2]. Attainment of the testing (92%) is close, but getting those tested onto treatment (72%) and virally suppressed (66%) remains a challenge [2]. Unlike many countries in the region, the majority (83%) of South Africa’s HIV treatment costs are funded domestically, and screening, diagnosis and treatment of HIV is largely provided in the public sector through the extensive primary health clinic network [3]. International donors such as The President’s Emergency Plan for AIDS Relief (PEPFAR) and the Global Fund to Fight AIDS, Tuberculosis and Malaria (Global Fund) contribute the balance of funding [4]. The donor funds are targeted for specific areas requiring additional resources with plans to ultimately transition these costs to the government. The current economy has forced the South African government to limit increases in budgets for certain services while the budgets for HIV and TB are projected to increase [4]. To ensure the sustainability and improve outcomes of the national HIV program, alternative sources of funding and service delivery need to be explored. One option to try and bridge the remaining gap between testing and treatment initiation is to leverage existing non-governmental primary health care providers (private providers) to further expand HIV treatment, and other services.

South Africa has a diverse mix of non-governmental providers who provide primary health care services, including HIV treatment. The traditional private sector general practitioner (GP) model of primary health provision caters largely for the 16% of South Africans who have private medical insurance through one of the 78 registered medical insurance schemes. However, nearly double this proportion (29%) of South Africans have said that the first health care facility that they would consult would be a private healthcare facility [5, 6]. This difference suggests that many uninsured individuals are seeking care outside of the public sector despite, not having medical insurance. The proposed National Health Insurance (NHI) system in South Africa plans to leverage the private sector to improve access to primary health care services for the entire population [7]. The shortage of GPs in South Africa suggests that the traditional GP centric model will not be sufficient to see to the needs of all South Africans, and new models of delivering primary health care services will be critical to the success of NHI- especially those targeted at those with limited or no income [8, 9]. We have reported previously on a number of innovative non-governmental models of primary health care delivery that aim to offer services to low-income population groups that are not insured [10]. There are however no cost estimates for the provision of these services.

To our knowledge, there is almost no work that describes the cost of private sector primary health care in South Africa as it pertains to the provision of HIV treatment [11]. If the South African HIV response is going to expand through the private sector, either with donor funding or through the implementation of the NHI, it is critical to understand the models of HIV treatment delivery being offered in the private sector. In particular the populations that the models serve, the costs associated with treatment using these models and the outcomes that patients achieve in these models. To address this, we estimate the resources used, costs and outcomes of HIV treatment across these models, and discuss differences between private and public-sector models.

## Methods

We conducted a cost outcomes analysis by collecting patient-level resource usage and treatment outcomes through retrospective medical record reviews and a bottom-up micro costing from the provider perspective (public or private payer). Data collection was conducted in 2019 and reflects services provided during the 4 years prior to that (2016–2019).

### Study sites

Private sector sites (Prv) were eligible if they were: a) a direct provider of primary health care (PHC) services (not a healthcare funder or managed care organization), b) offered a spectrum of PHC services that included at least one among HIV, TB, diabetes or hypertension treatment; c) focused primarily on low-income, uninsured populations and d) had a potentially sustainable model that was not completely reliant on donor funding. These were initially identified through purposeful sampling following desktop research and later expanded to include organizations identified through interviews with key informants. Further details of the models identified are outlined in an exploratory, descriptive study [10]. Those models that were currently active were asked to participate in the cost outcomes analysis. For this analysis, we focused on those providing HIV care and treatment.

Public sector PHC sites (Pub) were initially selected based on their physical proximity to the private sector PHC sites. However, two of the private sector clinic models had multiple physical sites and we used the entire dataset to reach the sample size. The public sector PHC sites included are all in Gauteng province and set in a similar urban setting to those of the private sector sites but are not necessarily in close physical proximity to all private sector sites. Setting was considered important as a proxy for the patient population that each site was serving. Public sector PHC facilities provide a full spectrum of PHC services and therefore were not selected based on service offering.

### Study population and sample

All adults (≥18 years old) living with HIV and seeking HIV treatment at the study sites were considered eligible if, at the time of data collection, they had initiated antiretroviral treatment (ART) or, if already on ART, presented to the site for the first time at least 15 months (12 + 3-month window) before start of data collection. The follow-up window was between January 2016 and December 2019. The study population was patients who started ART at the study site at least 15 months prior to data collection, which included both treatment naïve (i.e. patients who initiated at the study site) and ART experienced patients (i.e. patients presenting at the study site for the first time seeking continuation of HIV treatment). Any patient who formally transferred out of the study site during the 15-month follow-up period or who had a missing medical record was excluded. Patient records were then extracted starting with the most recent eligible patient and moving backwards until a sample of 75 patients per site was reached. The sample size was estimated based on prior cost estimates made at a typical primary health clinic in South Africa and was set to be able to detect a 10% difference in mean cost (alpha 0.05, power 80%).

### Data collection and analysis

Patient-level data included all resources used during a 12-month period to provide HIV treatment to the patient. These included medicines dispensed, laboratory tests performed, and visits made to the facility. This information was extracted from the patient medical records at the study sites. Some facilities had electronic medical records that allowed this to be extracted directly into the study database, while other sites used paper medical records and data capturers had to review and extract the data from these files into the study database. Patient outcome data was also obtained from the medical records. Patients were considered “not in care” if they died during the study period or had a gap in ART coverage > 90 days at 12 months. If patients were “in care” at 12 months, they were classified as responding based on the presence and value of a viral load. When these terms are used to reference these outcome definitions in the manuscript, quotation marks are used (i.e., “in care” or “not in care”). Viral load suppression was defined as having a viral load of ≤400 copies per ml between 9 and 15 months after enrollment. The 3-month window before and after 12 months allowed for variable timing in the routine 12-month viral load. The primary patient outcome at 12 months after enrollment was a composite measure based on retention “in care” and response to treatment (In care and responding). Secondary outcomes included; “in care” but not responding (retained but viral load > 400 copies per ml), “in care” with an unknown viral load (retained but viral load not reported) and “not in care” (not retained).

Costs were estimated from the provider perspective for the study period (12 months at the study site). For the public sector PHC facilities, variable costs were calculated by taking the individual level resource utilization (medicines dispensed, laboratory tests performed, visits) and multiplying it with the appropriate unit prices. We use two metrics to describe resource utilization, the ARV medicine possession ratio (MPR) and time in care. The ARV MPR measures the proportion of days in the 12-month period (365 days) that the patient was covered by antiretroviral (ARV) medication. Time in care measures the time from ARV treatment initiation at the study site or presentation at the site on treatment to the first of either the end of the follow up period (12 months) or the last day that they were covered by ART based on the last prescription filled in the study period. Fixed costs such as clinic space, administrative staff and utilities were estimated from site financial records and then allocated across the patient population, based on all patients’ visits at the respective study site. For the private sector models, the same approach was used unless the model was a fixed fee per visit model (study site Prv2 and Prv3) in which case the fee was used to reflect the cost to the government of offering public services through this model. If there were any additional resources that were not captured in this fee (medicines dispensed, laboratory tests performed), these were added to the model fee using the unit cost of that model. These costing methods are described by cost category in more detail in Table 1. The average cost per patient retained “in care” and virally suppressed is reported per model. The original cost estimates were reported in 2019 South African Rand (ZAR) and converted to 2019 United States Dollar (USD) using the mid-year exchange rate for the calendar year 2019 of 14:1 (ZAR:USD) [12]. Analysis of resource utilization and cost was done in Microsoft Excel (16.42) and Stata Statistical Software (Release 15).

**Table 1:** Costing methodology

Cost category	Estimation method
Medicines – ARV and non-ARV	All medicines (ARV and non-ARV) prescribed during the study period were extracted from the patient medical record. If at the last visit in the study period medicines were prescribed for a period that exceeded the study period then only the amount of those medicines necessary to cover the remaining study period were included. Drug costs were obtained from the 2019 Master Procurement Catalogue (National Department of Health, South Africa) national tender price list for sites that had access to public sector medicines (Pub1, Pub2, Prv1, Prv2) and from the South African Single Exit Price list (Medicine Price Register, www.mpr.org.za) for the site (Prv3) which procured privately.
Laboratory tests	All laboratory tests (HIV related and other) conducted during the study period were extracted from the patient medical record. Viral loads conducted after the study period (12–15 months) were used to assign outcome but not included as part of the resource usage. Laboratory costs for sites with access to state laboratory tests (Pub1, Pub2, Prv1, Prv2) were obtained from the National Health Laboratory Services State Price List (2017) inflated to 2019 prices using the annual inflation as reported by the IMF for South Africa [13]. We used laboratory costs from the private sector laboratory that serviced the site (Prv3) that did not have access to state laboratory costs.
Staff - clinical	All clinical visits to the site during the study period were extracted from the patient medical record. The salary costs of all staff providing clinical care were obtained from the site financial records or estimated based on the national salary scales published by the Department of Public Service and Administration for 2019 [14]. The cost per visit was estimated by taking the total annual salary cost for clinical staff and dividing it by the total annual patient visits. Each patient was then allocated a staff clinical cost based on the number of visits they made.
Staff – non-clinical	The salary costs of all staff who were not providing clinical care (i.e., administration, reception, social services) were obtained from the site financial records or estimated based on the national salary scales [14]. The cost per visit was estimated by taking the total annual salary cost for these staff members and dividing it by the total annual patients’ visits (i.e., visits for any reason). Each patient was then allocated a staff non-clinical cost based on the number of visits they made.
Fixed (Space, equipment, utilities, supplies)	The total space required to provide care was measured and the cost was estimated based on a market based rental estimate for the area. Equipment for the space was inventoried, valued at 2019 replacement cost and annualized over the estimated lifespan based on standard accounting guidelines. Utilities were estimated based on the size of the space and a standardized utility (i.e., power, water and refuse) estimate per square meter. Annual cost of non-medical supplies was obtained from facilities records and financial statements. The cost per visit was estimated by taking the total annual fixed costs and dividing by the total annual patient visits. Each patient was then allocated a fixed cost based on the number of visits they made.
Fixed fee	We used a fixed fee per patient visit for two of the private models (Prv2 and Prv3). This fee covered the fixed and staff related costs, as well as any network administration or management fees used in the model. Fees are determined based on the number of visits and services offered. Based on the resources utilized by each patient and the fee structure of the model, the fee for each patient was estimated. If government were to expand HIV services in the public sector through these models these would be the associated costs. Costs not covered by the fee such as medicines and laboratory costs are still reported for these models.

### Ethics approval

This was a retrospective record review without any direct patient interaction by the study team. The ethics boards of the University of the Witwatersrand and Boston University reviewed and approved the study protocols and provided a waiver of informed consent.

## Results

### Selection and description of models

The list of potential private sector PHC providers was finalized in August 2018. Of the seven distinct private models identified, six were active and offered HIV treatment in 2019, but only three were included in this study. Two were excluded because the necessary data were not available, and one was excluded because it was outside of Gauteng and primary data collection was not feasible. Table 2 is adapted from a previous publication [10] and provides a more detailed description of the models included and their characteristics. In the text and tables, the models will be referred to using the abbreviation listed in Table 2. Overall, we identified three innovative private models of primary health care provision that catered towards patients that could not afford medical insurance and provided HIV care and treatment, as well as two government primary health clinics that served patients from similar urban settings.

**Table 2:** Description of included models (Adapted from Girdwood et al. [10] Table 2)

Characteristic	Public 1	Public 2	Private 1	Private 2	Private 3
Abbreviation	Pub1	Pub2	Prv1 (NGO)	Prv2 (GP)	Prv3 (Nurse)
Model description	Nurse led; full-service primary health clinic run by the Department of Health (DoH).		Comprehensive PHC services provided by nurses and doctors in a large urban non-governmental organization. Largely integrated into the DoH.	A partnership between general practitioners (GPs), DoH and the private administrator. Private GPs were organized into an HIV disease management network and paid a capitated annual fee at the point of enrolling uninsured, treatment naïve patients who are not reached by the public sector.	Nurse-led ownership model. Clinics were owned and operated by a professional nurse and organized as an non-profit company and followed a franchise model with a network fee.
Target population	Served an urban population in Gauteng province seeking health care services in the public sector.	Served an urban population of a township in Gauteng province seeking health care services in the public sector.	The majority of beneficiaries were from surrounding informal settlements. A large proportion were foreign migrants. Most were employed but low income.	Employed, low income (‘the working poor’) in and around a Gauteng district. Aimed to target uninsured people living with HIV currently missed by the public sector owing to access barriers and who were cash-paying customers in the private sector.	Under-served communities. Employed (80%), unemployed (20%), i.e., elderly grant earners, but all low income and uninsured (LSM 1–4). Primarily targeted people seeking privacy, working mothers needing to vaccinate their children and STI treatment.
Financial model	Free at the point of care. All costs (medicines, laboratory services, visit costs) services were covered by the DoH.		Combination of private, donor and DoH funds (provided essential medicines, NHLS), as well as user fees to a small extent.	A donor funded the annual GP consult fee, dispensing fee and admin fee. DoH funded medicines, labs and test kits.	User fees covered operational costs. Upfront infrastructure funding and working capital donations (for first 2 years) provided by donors and National Treasury (Jobs Fund). Nurse required 250 patients per month to break even.
Volume^a^ / scalability	Standard government, primary health clinic. Served ~ 5100 patients per month.	Standard government, primary health clinic. Served ~ 6000 patients per month.	1 facility (and 1 mobile). Served ~ 7600 patients each month.	6 GPs currently contracted in one district (~ 640 patients during study period) but plans to expand nationally.	Currently has 41 sites but plans to expand to 70 by 2019. Each clinic served on average 1700 patients per month.

^a^Volume refers to total patient headcount and is not specific to patients living with HIV

### Patient characteristics

Overall, there were 376 patients enrolled across five facilities. Table 3 shows that in our sample the patient populations at the public sector sites were predominantly female (78 and 72%). In our sample, two of the private sector sites (Prv1 (NGO) and Prv2 (GP)) treated more men than the public sites, but the final private site (Prv3(Nurse)) treated more women than the public sites. The age distribution of patients was similar across sites, and almost all patients were on the standard fixed dose regimen of tenofovir-emtricitabine-efavirenz (TDF-FTC-EFV). The median baseline CD4 count at Prv1 (NGO) was 246 cells/mm^3^, while other sites that had median baseline CD4 counts above 300 cells/mm^3^. The majority of patients were ART naïve (Prv3 (Nurse) did not report this), and overall, approximately one tenth reported having either tuberculosis, hypertension or diabetes (mean number of comorbities, Table 3) when they started HIV treatment.

**Table 3:** Sample Characteristics

	Total		Pub1		Pub2		Prv1 (NGO)		Prv2 (GP)		Prv3 (Nurse)	
	N=376		n=76		n=75		n=75		n=75		n=75	
Gender, n(%)												
Male	177	(47%)	17	(22%)	21	(28%)	29	(39%)	35	(47%)	12	(16%)
Female	199	(53%)	59	(78%)	54	(72%)	46	(61%)	40	(53%)	63	(84%)
Age, n(%)												
18-30	111	(30%)	30	(39%)	24	(32%)	22	(29%)	17	(23%)	18	(24%)
31-40	151	(40%)	29	(38%)	24	(32%)	35	(47%)	27	(36%)	36	(48%)
41-50	77	(20%)	13	(17%)	16	(21%)	14	(19%)	19	(25%)	15	(20%)
>50	37	(10%)	4	(5%)	11	(15%)	4	(5%)	12	(16%)	6	(8%)
Age, median (IQR)	34	(29.0-42.0)	31.5	(27.0-37.5)	36	(29.0-45.0)	34	(30.0-35.0)	38	(27.0-29.0)	34	(30.0-40.0)
Baseline ART regimen, n (%)												
Fixed dose combination (TDF-FTC-EFV)	372	(99%)	75	(99%)	72	(96%)	75	(100%)	75	(100%)	75	(100%)
ABC-3TC-EFV	4	(1%)	1	(1%)	3	(4%)	0	(%)	0	(%)	0	(%)
Baseline CD4 count, n (%)												
<100 cells/mm3	42	(11%)	5	(7%)	14	(19%)	16	(21%)	7	(9%)	-	
101-200 cells/mm3	38	(10%)	5	(7%)	9	(12%)	10	(13%)	14	(19%)	-	
201-350 cells/mm3	73	(19%)	22	(29%)	14	(19%)	22	(29%)	15	(20%)	-	
>351 cells/mm3	124	(33%)	34	(45%)	29	(39%)	23	(31%)	38	(51%)	-	
Missing	99	(26%)	10	(13%)	9	(12%)	4	(5%)	1	(1%)	75	(100%)
Baseline CD4 count, median (IQR)	330	(166-509)	372	(272-496)	327	(116-513)	246	(115-410)	369	(191-664)	-	
ART naïve at enrolment, n (%)	293	(97%)	73	(96%)	71	(95%)	74	(99%)	75	(100%)	not reported	
Mean days on ART at enrolment, if not ART naïve	426		38		817		24		-		not reported	
Mean number of comorbidities	0.13		0.14		0.12		0.24		not reported		0.03	

### Patient-level HIV outcomes

Table 4 shows that there was a wide range (8–80%) in the proportion of clients that were classified as in HIV care at each of the sites at the end of the follow up period. In particular, we found that viral load testing, a component of our proposed primary outcome measure, was not conducted consistently across public and private sites with between 0% (0/75, Prv3) and 72% (55/76, Pub1) of patients having a recent routine viral load result in their file. Among those with a viral load test available, the majority (87%, 152/173) were suppressed. Across all the models those retained “in care” are the majority (> 50%) at 12 months, with the exception of Prv3 (Nurse) which reported only 8% “in care”. It should be noted that “in care” refers specifically to HIV treatment. The individual level data from Prv3 (Nurse) shows that some patients return to the clinic for other services, but choose not to access HIV treatment at these visits (not shown in table).

**Table 4:** Outcomes at 12 months and selected resource use

	Total		Pub1		Pub2		Prv1 (NGO)		Prv2 (GP)		Prv3 (Nurse)	
	N=376		n=76		n=75		n=75		n=75		n=75	
Outcomes, n (%)												
In care for HIV, all	227	(60%)	61	(80%)	55	(73%)	47	(63%)	58	(77%)	6	(8%)
In care & VL suppressed	152	(40%)	49	(64%)	25	(33%)	32	(43%)	46	(61%)	0	(%)
In care & VL unsuppressed	21	(6%)	6	(8%)	4	(5%)	5	(7%)	6	(8%)	0	(%)
In care & VL unknown	54	(14%)	6	(8%)	26	(35%)	10	(13%)	6	(8%)	6	(8%)
Not in care for HIV, all	149	(40%)	15	(20%)	20	(27%)	28	(37%)	17	(23%)	69	(92%)
Resource usage, mean (SD)												
ARV Medicine Possession Ratio	0.62	(0.37)	0.77	(0.28)	0.75	(0.33)	0.66	(0.39)	0.74	(0.31)	0.17	(0.15)
Time in care, days	247	(139)	299	(102)	288	(118)	252	(140)	301	(114)	93	(95)
Total visits	6.6	(3.8)	7.5	(3.0)	8.0	(3.3)	7.0	(3.9)	7.9	(3.4)	2.5	(2.1)
Total visits, per month in care	0.9	(0.4)	0.8	(0.2)	0.9	(0.2)	1.0	(0.3)	0.9	(0.4)	1.2	(0.8)
CD4 counts, number	0.7	(0.8)	0.8	(0.8)	0.2	(0.4)	0.9	(0.7)	1.3	(0.8)	0.2	(0.4)
Viral loads, number	0.8	(0.9)	1.3	(1.0)	1.0	(0.8)	0.9	(0.9)	0.8	(0.6)	0.0	(0.0)

### Resource utilization

The key resources utilized by the full sample during the 12-month period are summarized in Table 4. In general, all sites reported that on average their HIV clients were covered by ART for more than two thirds of the year, with the exception of Prv3 (Nurse) which reported a ARV MPR of only 17%. Time in care tracks very closely with MPR suggesting that for the time that patients were considered in care they were consistently on ARV treatment. During the study period all sites had an average of 0.8 visits per month (30 days) in care. CD4 count testing varied, but all sites had approximately 1 viral load in the 12-month period with the exception of Prv3 (Nurse) which did not have consistent laboratory testing reported.

### Patient and health system costs

Table 5 showed that the mean cost of patients “in care” are consistently higher than those “not in care”, which reflects the difference in time in care and subsequent resource usage. The NGO site (Prv1) showed costs consistently higher across the “in care” outcome categories, the majority of the difference comes from higher non variable costs, as shown in the cost breakdown by cost category for those “in care”. A major cost difference for this site was the large investment in staffing, in particular non-clinical support staff. All of the sites obtained drug and laboratory services through the government and so differences in drug and laboratory costs in Table 5 reflect differences in resource utilization; the exception is Prv3 (Nurse) which procured privately and had higher ARV costs. Overall, one private site (Prv2 (GP)) had a mean cost across all patients within the range (US$195-US$297) of the two public sector sites, while the other private sites were outside this range, one higher and one lower.

**Table 5:** Average cost per patient by outcome and cost category

	Pub1		Pub2		Prv1 (NGO)		Prv2 (GP)		Prv3 (Nurse)*	
	n=76		n=75		n=75		n=75		n=75	
Outcome, mean (SD)										
All patients	297	(115)	195	(86)	358	(200)	247	(85.29)	97	(71)
In care for HIV, all	335		238		489		287		206	
In care & VL suppressed	342	(78)	244	(53)	487	(112)	293	(29.52)	0	(0)
In care & VL unsuppressed	295	(116)	257	(8)	486	(51)	270	(40.93)	0	(0)
In care & VL unknown	319	(102)	230	(36)	497	(126)	261	(23.23)	206	(96)
Not in care for HIV, all	142	(96)	76	(51)	138	(95)	110	(65.18)	88	(60)
Cost category for in care, mean (%)										
Drugs - ARV	90	(27%)	94	(39%)	101	(21%)	87	(30%)	144	(70%)
Drugs - non ARV	16	(5%)	5	(2%)	19	(4%)	17	(6%)	4	(2%)
Laboratory tests	59	(18%)	39	(16%)	65	(13%)	48	(17%)	0	(%)
Non variable costs	170	(51%)	100	(42%)	305	(62%)	136	(47%)	58	(28%)
Staff - Clinical	113	(34%)	62	(26%)	117	(24%)	0	(0%)	0	(0%)
Staff - Non clinical	17	(5%)	23	(10%)	108	(22%)	0	(0%)	0	(0%)
Fixed costs	40	(12%)	15	(6%)	79	(16%)	0	(0%)	0	(0%)
Fixed fee**	0	(0%)	0	(0%)	0	(0%)	136	(47%)	58	(28%)
Total	335	(100%)	238	(100%)	489	(100%)	287	(100%)	206	(100%)
Summary costs for in care										
Total cost per month in care, mean (SD)	31	(7)	20	(4)	47	(13)	29	(14)	44	(21)
Non variable cost per visit	20		10		32		17		13	

*Medicines and labs procured at private sector prices

**Fixed fee includes fixed costs and staff costs for these models; paid by donors in Prv2 and by patients in Prv3

## Discussion

The results presented from this cost outcomes study provide some evidence to support the possibility of expanding primary health care services, in particular HIV treatment, beyond the traditional government-owned and -run primary health care facilities. We see that across the three different private models presented, there are differences in the costs and outcomes associated with HIV treatment, but at least one of the models is within the range of the primary health clinic results that we report. The nurse-led model (Prv3) appeared to be distinct from the others (Prv1 (NGO) and Prv2 (GP)). The differences reported across the private models might be partially explained by differences in the patients accessing care and treatment at these facilities, which are not captured by the basic demographics data we have from medical records. In patient interviews that were carried out across public and private primary health care sites, including our study sites, some differences were reported [15]. Namely that patients accessing primary health care privately had a higher average income and were more likely to be male compared to those who sought services exclusively in the public sector or used a mix of public and private. Despite these differences the reported prevalence of a chronic condition (tuberculosis, diabetes or hypertension) was similar across patients accessing services across public and private study sites as reported by Govender and colleagues [15].

The variation in the sample population and the differences in outcomes and costs across models indicates that it would be incorrect to treat public primary health clinics and private delivery models as homogenous groups, respectively. There appear to be two clear distinctions between the private sector models presented which may also contribute to the results we see. Two of the private models (Prv1 (NGO) and Prv2 (GP)) had access to government-procured and -supplied antiretroviral medicines and laboratory tests, while the nurse-led model (Prv3) accessed both commodities through the private sector. In addition to this, the nurse-led model is the only model that charges a patient fee for each consultation and service provided aimed at full cost recovery. The NGO model charges a nominal user fee per visit (excluding the first visit which is free to patients), but it is lower and designed to cover only administration costs. It is possible that patients in the Prv3 (Nurse) model manage their HIV treatment through a combination of public sector and private sector care. In the individual level data from Prv3 (Nurse), it is seen that some individuals come back during the 12 month follow up period for other services, but not consistently for HIV treatment. The targeted use of private sector health care for certain conditions was reported by Grépin who showed, using Demographic and Health Surveys from 70 low and middle income countries, that care for a child’s diarrhea or fever was likely to be sought in the private sector while care for child birth, ante natal care and contraception was sought predominantly in the public sector [16]. In our study it may be that individuals are using the convenient, easily accessible services offered by this nurse model for acute services when they have the resources, but that regular chronic care through this model is too expensive for this population. This is supported by qualitative work done across public and private sector primary health care sites (including the study sites) where it was noted that patients at private clinics were more likely to report seeking acute care compared to those at public sector clinics [15]. The GP contracted model (Prv2) was the only private sector model that focused specifically on HIV treatment exclusively, which partially explains why the costs are so different from the NGO (Prv1) model which provides a full primary health service model to its patients.

Given these significant differences in the private sector delivery models that have implications for both costs and outcomes, we should not draw direct comparisons between individual private sector models, but rather understand how each model can be leveraged in conjunction with the current public sector PHC models to attain the UNAIDS goals within this resource limited setting. Previous systematic reviews that examined the provision of ambulatory care offered by public and private providers in low- and middle- income countries have drawn different conclusions [17, 18]. One reason for this stems from the lack of data in this area but also the challenge of comparing primary care across conditions and diseases [19]. A strength of this study was the primary data collection and the focus on HIV treatment provision, which provided us with a “standard patient”. However, even within the pool of people living with HIV, we note likely heterogeneity in patient characteristics across models. With all the models presented it is likely that the outcomes and costs will vary depending on the patient population that accesses care through them. Patients’ ability to access care through a model is likely to be closely linked to the cost to the patient as it has been shown that user fees act as a barrier to access [20]. Except for the nurse-led private sector model (Prv3), none of the models charged any significant user fees. The fact that some patients were willing to pay for occasional services suggests that they see value in the service and that if the user fee were covered by NHI patient outcomes and costs may look very different for this model. A report investigating the potential of using the private health sector to expand the HIV/AIDS workforce also acknowledges how critical the mechanism for paying the private sector would be in its success in expanding HIV services in resource limited settings [21]. A recent evidence gap map highlighted how data to support the role of contracting out to provide primary health care services and how to govern this contracting process are still missing for the low and middle income country setting [22]. The models reported here offer a potential approach to alleviate some of the burden off the straining public health infrastructure and are examples of models that could be contracted through NHI to provide healthcare services to the broader public.

This study has several limitations. Only three distinct private sector delivery models were included, and there are other variations of these models in South Africa now. These results should be used to understand the possible drivers of differences the cost per outcome in these models, but it may be inappropriate to generalize the results directly to other sites. The sample sizes were modest, and certain outcome categories became very small when calculating the average cost per category. The study data was reliant on standard patient medical records at each of the sites, which meant that data availability, which was not consistent across sites, in part drove outcome assessments. For example, we do not know the number of comorbidities for site Prv2 or the baseline CD4 count or ART naïve status at Prv3. The study deliberately took the perspective of the Department of Health and what their costs might be to expand services using these models; as a result, it specifically excluded the costs to the patients of accessing care (i.e., time, transport) through these models. Differential patient costs combined with the user fees may be a factor in explaining why certain populations (e.g., male versus female) were drawn disproportionately to certain models. Lastly, we have hypothesized that differences in patients accessing care may partly explain why there were differences in the outcomes across models, but the data presented here is not sufficient to fully explain why these outcomes differed across models except for baseline CD4 count and how that may influence treatment success. Ideally, we would have liked to understand some of the socio-economic characteristics of the patients accessing these models, but that was not available in the routine medical data.

## Conclusions / implications

Despite these limitations, the results presented here provide some of the first evidence showing the cost associated with providing treatment to uninsured, low income patients outside of the traditional primary health clinics in South Africa using three distinct models. For these models it appears that requiring patients to pay for services (Prv3 (Nurse)) may result in less consistent retention for HIV treatment, while providing services with limited or no cost to patient at the point of care (Prv1 (NGO) and Prv2 (GP)) provided outcomes similar to those found in the public sector. Costs in the private models are similar to those found in the public sector sites, but sites that offered comprehensive services (Prv1 (NGO)) in addition to HIV care and treatment had higher costs. The results suggest that offering HIV treatment under NHI through delivery models similar to these may be able to expand access with similar outcomes and costs to those currently found in the public sector. Further research is required to understand how removing patient costs at the point of care as planned under the NHI might change the outcomes seen here.

## Data Availability

The patient level data that support the findings of this study are owned by the National Department of Health and the private health care providers who participated in the study, and so are not publicly available. Data are however available from the authors upon reasonable request and with permission of the National Department of Health (South Africa) and private health care providers.
